# The Generation Process, Impurity Removal and High-Value Utilization of Phosphogypsum Material

**DOI:** 10.3390/nano12173021

**Published:** 2022-08-31

**Authors:** Xinfeng Lv, Lan Xiang

**Affiliations:** Department of Chemical Engineering, Tsinghua University, Beijing 100084, China

**Keywords:** phosphogypsum material, generation process, impurity removal, high-value utilization, Nano-CaCO_3_, Nano-calcium sulfate whisker

## Abstract

As phosphogypsum constitutes a large amount of solid waste material, its purification treatment and comprehensive utilization have close connection with economic development and ecological environmental protection. For the moment, the storage quantity of phosphogypsum is still rising as a result of the increasing phosphate fertilizer production to meet the food demand in China. This paper summarizes the generation process, impurity removal treatment (physical method, chemical method, heat method), high-value utilization (nanometer calcium sulfate whisker, nanometer calcium carbonate) of phosphogypsum material and some existing problems. It puts forward some views on the challenges in this field and the direction of future development. It is hoped that the investigation and summary in this paper could supply some significant information for the impurity removal and high-value utilization of phosphogypsum material as a contribution to sustainability.

## 1. Introduction

China is a large country and rich in calcium resources, with abundant reserves of natural gypsum and secondary gypsum resources [1]. Natural gypsum is mainly gypsum ore, which is divided into dihydrate gypsum ore (CaSO_4_·2H_2_O) and anhydrite ore (CaSO_4_) according to the chemical composition. The natural gypsum is mainly used as a cement manufacturing retarder [1,2,3], while secondary gypsum (industrial by-product gypsum) is a typical representative of solid waste material.

**Figure 1:**
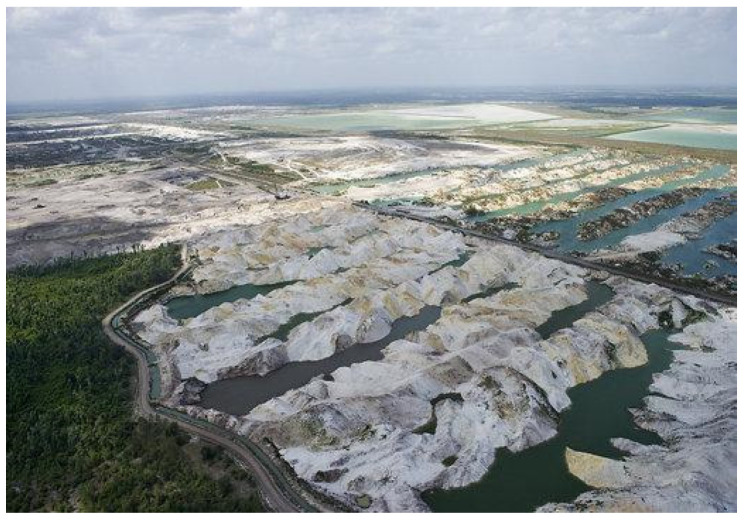
The storage condition of PG in Florida.

**Figure 2:**
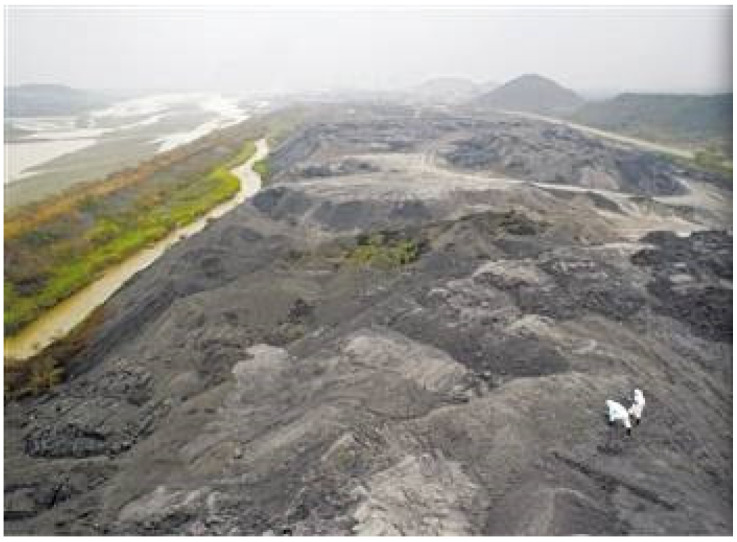
The storage condition of PG in China.

Phosphogypsum (PG) represents the typical secondary gypsum material; its main composition is also of dihydrate calcium sulphate. At present, the world’s PG pile stock has reached 6 billion tons, and is still being added at the rate of 150 million tons every year. China is the world’s largest producer of phosphate fertilizer, and also the largest producer of PG as a by-product. With the development of the phosphate fertilizer industry, PG production increased year by year, but most is still in open storage. The stock of PG has reached 400 million tons, and is still increasing by about 50 million tons every year, leading to increasingly serious environmental problems and hidden trouble; more dam and environmental pollution incidents have happened [4,5,6,7]. The storage conditions of PG in Florida and China are shown in Figure 1 and Figure 2.

In the process of PG storage, various impurities and ions soaked by rain and snow will cause pollution to the soil, surface and groundwater, so it is necessary to treat the seepage wastewater [5]. In addition, in accordance with the “environmental protection tax law”, China has levied a tax on solid waste (25 yuan/ton) since 2018, and no new storage sites have been approved. Many areas in southwest China have even “decided production by slag”. The sustainable development of chemical enterprises is facing great environmental pressure [8,9,10]. Therefore, the impurity removal and high value utilization of PG are increasingly considered by researchers.

## 2. Generation and Composition of PG

PG results from the process of wet phosphoric acid production from the reaction of phosphate rock and sulfuric acid with CaSO_4_·2H_2_O as the main composition of the byproduct, while it contains a small amount of other impurities, such as phosphorus, fluoride and organic matters [11]. As to the wet phosphoric acid production process, phosphate ore is decomposed by sulfuric acid to produce extraction slurry, and then phosphoric acid is filtered. Meanwhile PG is also produced. The main reaction equation is as follows [12]:

Ca_5_F(PO_4_)_3_ + 5H_2_SO_4_ + 10H_2_O = 5CaSO_4_·2H_2_O + 3H_3_PO_4_ + HF↑ (1)

For every 1.0 t phosphoric acid produced, 4.0–5.5 t PG is produced [13,14,15,16,17]. At present, the annual production of PG in the world is up to 200 million tons, and about 70 million tons in China. The emission of PG is increasing year by year with the rapid development of high-efficiency compound fertilizer industries. Currently, about 150 million tons of PG have been accumulated in China, most of which are in an idle state. The comprehensive utilization rate of PG is only 40%. Because of the PG containing phosphorus, fluorine and more harmful impurities, arbitrary discharge and the long-term accumulation can cause the pollution of surface water and groundwater. This is a major environmental problem [18,19] and, therefore, it is vital to research the impurity removal and high value utilization of PG.

The main chemical composition of PG is basically the same as that of natural gypsum, which also contains P_2_O_5_, SiO_2_, Al_2_O_3_, Fe_2_O_3_, organic compounds and a small amount of cadmium, lead, sodium and other elements [20,21,22]. The chemical composition of PG in different production enterprises and batches is slightly different, which is mainly related to the control of phosphoric acid production process conditions and the variety of phosphate ore. PG has higher water content, and free water content is as high as 15–20%. and the pH is about 4.5–5.5. PG shows dispersive fine grain and the aged PG mostly appears off-white, sometimes earthen yellow; the whiteness is about 30–55 [22]. PG mainly exists in the form of plate crystal, polycrystalline crystal, dense crystal and acicular crystal [23,24]. In the process of wet process phosphoric acid production, some phosphorus ores are not decomposed, PG washing is not complete, and isomorphic substitution is the main reason that PG contains phosphorus, silicon and other impurities. The presence of impurities makes the performance of PG inferior to natural gypsum [25] and, therefore, it cannot be directly applied to the production of gypsum building materials and high-end gypsum products. Therefore, it is vital to pretreat PG to remove impurities before high value utilization can be realized.

## 3. Classification and Existing State of Impurities in PG

Before removing impurities from PG, we first need to understand the specific nature of the various impurities. Researchers have classified the impurities according to their effect on the properties of PG. The following sections describe the phosphorus impurities, fluorine impurities, organic impurities and so on.

### 3.1. Phosphorus Impurities

Phosphorus in PG mainly exists in three forms: soluble phosphorus, eutectic phosphorus and insoluble phosphorus [26]. The soluble phosphorus has the greatest effect on the properties of PG. Soluble phosphorus impurities are formed by adsorption. The soluble phosphorus in PG distributes on the surface of dihydrate gypsum crystal and is adsorbed by dihydrate gypsum [27]. This kind of adsorption is mainly physical adsorption, and the adsorption force is surface force. Soluble P_2_O_5_ exists in PG in the form of H_3_PO_4_ and its corresponding salt, and its distribution mainly depends on the pH value in the hydration process. The acidity is mainly H_3_PO_4_ and H_2_PO_4_^−^, while the alkalinity is mainly in the form of PO_4_^3−^. Since the Ca^2+^ concentration in gypsum is relatively high, while Ca_3_(PO_4_)_2_ is an insoluble salt with low solubility, the PO_4_^3-^ concentration in the system is relatively low [28], so the soluble phosphorus in PG mainly exists in three forms: H_3_PO_4_, H_2_PO_4_^−^ and HPO_4_^2−^. Eutectic phosphorus impurities are formed by doping. Because CaHPO_4_·2H_2_O and CaSO_4_·2H_2_O belong to the monoclinic system, and are relatively close in lattice constant, so under certain conditions, CaHPO_4_·2H_2_O can enter into the lattice of CaSO_4_·2H_2_O to form a solid solution [29]. In this case, the different components can mix on the atomic scale, without damaging the host crystal phase structure, with only a little change in terms of crystal cell parameters, called eutectic of phosphorus. Eutectic phosphorus enters the lattice and cannot be removed by ordinary physical and chemical treatment. When semi-hydrous gypsum hydrates, eutectic phosphorus is released, and under the action of Ca^2+^ in the solution, insoluble Ca_3_(PO_4_)_2_ is formed to cover the surface of gypsum crystal, which delays the setting and hardening of gypsum and reduces the early strength of the hardened body [30]. So far the influence of eutectic phosphorus has been recognized by the academic researches, but there are only a few reports on its determination and analysis. Insoluble phosphorus exists in a small amount of unreacted phosphorus lime powder, and the composition mainly is Ca_3_(PO_4_)_2_. As an inert component, insoluble phosphorus exists in coarse PG and has no adverse effect on the properties of PG [31]. 

### 3.2. Fluorine Impurities

The fluorine in PG comes from phosphate ore. When phosphate ore is decomposed by sulfuric acid, 20~40% of the fluorine in phosphate ore is left in PG, in the form of soluble fluorine (NaF) and insoluble fluorine (CaF_2_, Na_2_SiF_6_, Na_2_AIF_6_) [32]. Soluble fluorine is adsorbed on the surface of gypsum crystal, mainly under the action of surface force, which is the main form affecting the performance of PG. It has a coagulative effect [18]. If the soluble fluorine content is low, it has little influence on the performance of PG, but when its mass fraction exceeds 0.3%, it will make the hydration product crystal coarsen, with the molecular force between the crystals weakened and the structure loose, thereby reducing the strength of PG. The insoluble fluorine is inert and can be used as an inert filler having no effect on PG.

### 3.3. Organic Impurities

The organic matter in PG comes from the organic impurities in phosphate ore and the organic additives added in some processes [33]. The organic matter is generally flocculent and adsorbed on the crystal surface of dihydrate gypsum. It mainly includes ethylene glycol methyl ether acetate, isothiocyano-methane, 3 mono-methoxy n-pentane, etc. When PG is used, the presence of organic matter will obviously increase the amount of water required; at the same time, it will weaken the joint between the crystals of gypsum dihydrate andweaken the molecular force between the crystals, so that the structure of the hardened body is loose and the strength is reduced [34].

## 4. Removal of Impurities in the PG

There are two basic ways to remove impurities in PG: two-step acidolysis based on source removal and direct removal based on PG post-treatment.

### 4.1. The Source Removal Method of Two-Step Acidolysis

As to PG source removal, high purity gypsum can be prepared by two-step acid hydrolysis. The principle of two-step acidolysis is [35]: react phosphate with hydrochloric acid or nitric acid to produce phosphoric acid and calcium into the solution in the form of calcium chloride or calcium nitrate:

5Ca_5_F(PO_4_)_3_ + 10HCl = 5CaCl_2_ + 3H_3_PO_4_ + HF↑ (2)

5Ca_5_F(PO_4_)_3_ + 10HNO_3_ = 5Ca(NO_3_)_2_ + 3H_3_PO_4_ + HF↑ (3)

The main problems of this method, which is still in the research and development phase, are: the main process of phosphate decomposition needs to be changed, the high-temperature acid volatilization process is harsh and the energy consumption is high.

### 4.2. The Direct Removal Method of Post-Treatment

As to PG post-treatment method, it does not change the status quo of production of wet process phosphoric acid, which is more easily accepted by industry, so it is becoming a research and development hotspot. Tayibi et al. [36] depicted that PG post-treatment methods mainly include physical, chemical and heat treatment methods.

#### 4.2.1. Physical Methods

The physical treatment method is a method to remove soluble impurities and organic matter. Because the physical method mainly relies on mechanical movement, washing, stirring and other processes, through the physical interaction to remove impurities, it does not involve chemical reactions. For soluble impurities and organics, most of them are adsorbed on the surface of PG through the interaction of surface forces, which can be removed by physical method. Physical methods to remove impurities include water washing, flotation, ball milling, screening and aging [33].

Among the methods of physical decontamination, washing is the most effective. Washing can remove most of the soluble impurities, but in terms of the environment, the sewage after washing must be treated before discharge or reuse, otherwise it will cause secondary pollution. The flotation method is a kind of wet treatment, letting water and PG with a certain ratio into the flotation equipment; after stirring and standing, organic matter and some other impurities can be removed. The advantage of this method is mainly that the water can be recycled and can be better if used in conjunction with other processes [37]. The ball mill method is an effective way to change the structure of PG, can make the PG particle morphology columnar, tabular, granular and so on, and can change the particles from the normal distribution to diffuse distribution. However, the ball milling method does not eliminate the adverse effects of impurities [38]. If it can also be combined with other impurity removal methods, the effect will be better. In addition, physical methods such as screening and aging are not commonly used. The screening method can only be used when the content of impurities is particularly high in a small range, and after screening, the corresponding pretreatment methods should be used for the removal of impurities with different particle sizes [39]. Finally, the aging method is a very simple way to deal with PG. It is to pile up PG and let volatile substances volatilize naturally with removal by natural wind. Nevertheless, the disadvantage is also evident, because if the aging is short-term, the effect will not be obvious; only if the aging time is extended, the aging effect will be positive, but the surrounding environment will be polluted with the exudation of impurities in PG.

Peng Jiahui et al. [40] carried out a systematic comparative study on various physical impurity removal methods of PG. The results showed that washing was the most effective way to remove impurities. The main reason was that this method was easy to operate and it was easy to remove a lot of soluble impurities by destroying the PG surface weak surface force of absorption; the disadvantages of washing were that the process investment was large and the energy consumption was high, and the problem of secondary pollution needed to be solved. Manjit Singh et al. [13] studied the removal of impurities in PG by wet sieve swirl. The process was based on wet screening according to the size of the particles, using a hydraulic agitator to pass PG through a 300-micron sieve. The experiment showed that the PG with the particle size of 10~15 microns was dissolved, and the organic matter, fluoride and phosphate on the crystal surface and some other impurities in the crystal lattice were also washed away successively. In this process, the impurities in the lattice were partially removed, which was considered to be caused by the effect of the hydraulic agitator on the crystal structure of PG; some impurities in the lattice dissolved, so it could be seen that the PG could be purified better by this process. El-Didamony et al. [41] found that after taking the method of physical extraction by selecting the appropriate organic extractor, Pb210, Ra226, K40 and U238 in PG could remove to the extent of 76.4%, 71.1%, 75.7% and 62.4% of their total content. This was mainly to consider that these elements could have a good solubility in the organic extraction agent, so as to achieve the removal of rare earth elements in PG by extraction. Wen Mingshu [42] adopted a flotation method to remove impurities from Yunnan PG. Experiments showed that this method not only removed the impurities of oil and organic matter, but also removed the water-soluble impurities P_2_O_5_ and fluorine, and achieved a removal rate of SiO_2_ of 80%, giving a better purified PG better. Koopman and Witkamp [43] described that 90% of the heavy metals and lanthanides found in PG could be removed by recrystallization and the application of exchange or membrane techniques. In this study, according to the principle that recrystallization utilized different substances with different solubility at different temperatures, the solubility of the heavy metals and lanthanides in the solid mixture of PG varied significantly with the temperature; the solubility was large at higher temperature, but small at lower temperature, enabling separation and purification. Bai Youxian et al. [44] studied the technological conditions for the removal of impurities in PG by washing. The experimental results showed that the optimal washing process conditions were as follows: the mass ratio of liquid to solid volume was 3 mL/g, and the washing process was conducted 3 times at room temperature. In this process, water could be recycled, and after three washings, the washing efficiency reached 96.68%. The P_2_O_5_ mass fraction in PG was less than 0.1%. The subsequent process also recycled P_2_O_5_.

#### 4.2.2. Chemical Methods

The chemical treatment of PG is to add certain chemical substances to PG, so that the impurities can be completely transformed into other precipitation substances or soluble compounds after chemical reaction; this can be used to treat PG with stable quality and low organic matter content [45]. For example, adding alkaline substances such as hydrated lime, quicklime, or acid substances such as citric acid, sulfuric acid, nitric acid to PG, to change the pH of the PG system, eliminates the influence of some impurities on its performance; at the same time, it can make some soluble matter into inert matter, reducing its adverse effect on the performance of PG.

After carrying out a series of researches, Yang Peihao proved that lime neutralization for PG could not eliminate the influence of organic matter on its material properties [46]. Therefore, the lime neutralization method was suitable for PG with low organic matter content. This method mainly eliminated the influence of acidic impurities through the principle of acid-base neutralization reactions. Potgieter et al. [47] used different substances to wash PG such as 1% H_2_SO_4_, 1% HCl, 10% NH_4_OH, 5% NaOH or milk of lime in order to remove the impurities of PG. After comparison, the effect of milk of lime was best. Mun et al. [48] used 0.5% milk of lime to wash raw PG for 5 min at 20 °C. The ratio of PG/milk of lime of 14% achieved the best effect. The results showed that the removal rate of impurities decreased and fluctuated with the increase of solid content, which made stirring more difficult, and reduced liquid solid contact. After neutralization treatment, PG was dried at 80 °C. Bai Youxian et al. [49] used a sulfuric acid pickling method to remove impurities from PG, studied the reaction conditions of this process, and finally obtained a high-quality PG. The experiment showed that under the conditions of sulfuric acid mass fraction of 35%, reaction temperature of 60 °C, reaction time of 4.5 h and liquid-solid mass ratio of 3 mL/g, the mass fraction of P_2_O_5_ in PG could be reduced to less than 0.01%. Al-Hwaiti [50] used various substances to treat PG by washing with solutions such as tap water and lime water, seawater and lime water, distilled water and lime water, tap water and 5% sulfuric acid, seawater and 5% sulfuric acid, distilled water and 5% sulfuric acid, limewater and 5% sulfuric acid and so on. Finally, it was found that the impurity removal effect on PG was best using the limewater and 5% sulfuric acid environment, considering that the limewater and sulfuric acid promoted each other when they played their roles. By adjusting the ratio of the two, the pH of the system was optimized, and the acid and base reacted with impurities at the same time, removing impurities more thoroughly. Singh et al. [51] added PG samples to 5–20% ammonium hydroxide aqueous solution oscillation, shaking well and then standing for 24 h under 35 °C. The sample was cleaned with 0.5% ammonium hydroxide solution first, and then was washed with water, and finally was dried under 42 °C. The analysis results showed that by using this method for processing PG, all impurities were reduced to a certain extent. Thus, it was proved that ammonium hydroxide could react with many impurities in PG, leaving the surface of PG and entering the solution. Meanwhile, organic impurities on the surface of PG could also be dissolved under the action of ammonium hydroxide, improving the whiteness of PG. Manjit Singh et al. [45,52] purified PG with citric acid. They removed the impurities from PG by shaking it with 2–5% aqueous citric acid solution for 15–25 min at 30 °C in mechanical shaker. The new product was washed with 0.5–1% aqueous citric acid solution, then washed with water and dried. Finally, they found that phosphorus and fluorine and other impurities could be converted into citrate, aluminate and ferrate that could be washed, so the aim of removing PG impurities was realized. Ölmez and Erdem [53] refined PG by washing with water first, then mixing milk of lime with it and washing with milk of lime for 5 min at 20 °C after calcination at various temperatures to determine the optimal impurity removal condition. Yang Min et al. [54] pretreated PG with lime solution, ammonia solution and citric acid solution of different concentrations. The results showed that when the PG treated under different conditions was added into cement as retarder, the retarder made of PG after removing impurities with citric acid had the best effect, considering that the damage to PG structure was minimal in the citric acid environment. Aly and Mohammed [55] removed fluoride and lanthanide from PG by HNO_3_ and NaNO_3_ as well as tributyl phosphate, using combined acid removal and physical extraction. The lanthanide elements had a good solubility in tributyl phosphate, and the removal rate of fluoride in PG was up to 80% after being treated with HNO_3_ and NaNO_3._ Leaching was not complete, which might be related to the dissolution and transformation of PG in HNO_3_. After characterization, it was found that the basic morphology of PG was still retained after the leaching treatment, and only the particle size and surface characteristics were changed, so it could be speculated that the fluorine in the crystal lattice of PG was not completely leached. Van der Merwe and Strydom [56] performed experiments in which PG was well stirred in sulfuric acid for 30 min at 25 °C, at a solid/liquid ratio of 1/4. At first, the removal rate of fluorine increased with the soaking time; however, at the soaking time of 80 min, the removal rate of fluorine suddenly decreased by about 3%, which was considered to be related to the crystallization change of PG. During this period, when the removal rate of phosphorus impurities in PG was investigated, a phenomenon of sudden increase was found, which may be caused by the crystal form transformation interference of PG.

Through the summary of the above research, it can be seen that the selection of various chemical substances and the determination of various treatment conditions are very important in the process of removing impurities of PG by chemical methods.

#### 4.2.3. Heat Treatment Methods

The method of heat treatment is to calcine PG at high temperature to remove impurities. The principle of this method is that impurities such as soluble phosphorus and eutectic phosphorus can be converted into inert pyrophosphate by calcining, while organic matter can volatilize. If the traditional heating method is adopted, the influence of inorganic substances can be eliminated only when the temperature reaches 800 °C. At this point, the content of eutectic phosphorus in PG is zero, so calcination is suitable to remove impurities from PG with high content of organics and eutectic phosphorus [57].

There were multiple studies which tried to remove the impurities from PG by calcination, and to determine the optimum calcination temperature. Singh et al. [58] calcined PG at 800 °C for 4 h. Smadi et al. [59] calcined PG at temperatures of 170, 600, 750, 850 and 950 °C for 3 h. Singh and Garg [60] calcined PG at temperatures ranging from 500 to 1000 °C with a step of 100 °C. Leskeviciene and Nizeviciene [61] calcined PG at 800 and 900 °C for 30 min. Rashad [19] calcined PG at 850 °C for 2 h. The results showed that the optimum calcination temperatures were in the range of 750–850 °C. The basic law of the calcination process was that with the increase of calcining temperature, the soluble impurity content in PG significantly decreased. This was because the soluble impurity part was volatilized, or converted into inert insoluble substance, so that its content was greatly reduced. Especially when the temperature was raised to 800 °C, the contents of soluble phosphorus and soluble fluorine were very low, the soluble phosphorus content was 0.06% and the soluble fluorine content was less than 0.01%.

With another focus, Yang and Qian [62] calcined PG at 500 °C for 4 h. Garg et al. [63] calcined PG at 150 °C for 4 h. Singh [64] calcined PG at 160 °C for 4 h. Singh and Garg [65] calcined PG at 750 °C for 4 h. Taher [20] calcined Egyptian PG at temperatures of 200, 400, 600 and 800 °C for 2 h. At the same time as exploring the optimal temperature of roasting and impurity removal, they were also committed to improving the performance of PG as tailings. The experimental results showed that the pre heat treatment of PG could effectively improve the hydraulic properties of slag cement, and the best hydraulic properties of slag cement were from PG produced after 800 °C heat treatment.

Considering that the traditional heating method had a large energy consumption cost, and the application would be very limited, the researchers further studied the removal of impurities in PG by microwave heating and irradiation, and found that the microwave heating was very fast, uniform and sensitive. Xu-dong Hu et al. [66] purified PG by a microwave heating processing method. The experimental results showed that the microwave calcination treatment could quickly remove the free water, organic matter and other impurities from PG. Compared to the traditional heat treatment methods, this was a new type of pollution-free mode of heat treatment and potentially energy-saving. Adrian Lambert et al. [67] removed rare earth elements from PG by microwave pretreatment. They found that 80% Nd, 99% Y, and 99% Dy leaching efficiency from PG was achieved at the optimum microwaving conditions of 15 min irradiation (2.45 GHz) at 1200 W. The effects were caused by changes in the microstructure and mineralogy of the PG. Reid et al. [68] demonstrated that during microwave pretreatment, the mineral water was heated and vaporized, which could cause nanosized cracks and pores in the mineral, allowing enhanced infiltration of leaching agent into the particle matrix and facilitating the extraction of elements from PG. The PG morphology had a nonlinear progression with microwave irradiation time, which was due to the factor that the drying rate of microwaved gypsum depended primarily on the particle moisture content (a nonlinear trend) and the microwave power [69]. It was observed that, as the microwave irradiation duration increased, the PG morphology had changed gradually, resulting in increased surface roughness and particle porosity [68]. In contrast, there were no morphological changes when the PG samples were heated in a conventional oven at 100 or 150 °C. The research of Yang et al. [70] had shown that microwave irradiation could induce the formation of CaSO_4_·0.5H_2_O, which required temperatures exceeding 107 °C, by creating localized hot spots. In the study of Lindroth et al., when a sample of wet gypsum was exposed to 1400 W for 2 min, a small amount of CaSO_4_·0.5H_2_O was produced, with no CaSO_4_ production [71]. In the study of Adrian Lambert et al. [68], as the microwave power and duration was increased, there was a sharp drop in CaSO_4_·2H_2_O content, accompanied by a proportional increase in CaSO_4_·0.5H_2_O and CaSO_4_. Thus, it could be seen that microwave heating and irradiation caused a distinct change in the crystal structure of the PG sample, causing a shift from the doubly hydrated gypsum to hemihydrated gypsum to anhydrous gypsum. Therefore, in the process of the removal of impurities in PG by microwave heating, the changes in microstructure caused by the phase conversions were a significant factor, especially for REE extraction; at the same time, nanosized cracks and pores in PG made it more permeable to leaching agents.

From what has been discussed above, microwave heating and irradiation, as a complementary technique, can be adapted to enhance a wide variety of PG impurity removal processes, which has a exceptionally promising prospect of application due to presenting a considerable cost saving opportunity. However, the technique also has the problems of potential scale-up complications and increased process complexity [68], and requires further research.

## 5. High-Value Utilization of PG Material

In consideration of the composition characteristics of PG (more than 90% is CaSO_4_.2H_2_O) and its attractive economic potential, and of continuously increasing concerns about environmental pollution as well, currently there is a great interest in using PG after the removal of impurities as an alternative raw material for various applications. Some researches described that PG had been used for soil stabilizers or as an agricultural fertilizer [72,73,74], and as a fly ash–lime reaction activator with wide application in the manufacturing of building materials [75]. Akın and Yesim described that PG had taken the place of natural gypsum used in the cement industry as a setting regulator [76] and in the gypsum industry to make gypsum plaster. According to the previous researchers, the total use of PG in building materials was probably well below 15% of the world PG production [77]. In the United States this use was discontinued in 1990 [78] and in 1992 it was also banned in the European Union. At present, the main way of comprehensive utilization of PG in China is still to prepare gypsum-based building materials [79,80,81,82,83,84]. For example, PG was used for building materials, notably gypsum board and gypsum wall material. The principle is to heat and decompose the calcium sulfate dihydrate to form the semi hydrated calcium sulfate, the latter forming the high-strength calcium sulfate dihydrate hardening material through hydration and cementation.

For the moment, although the technology of using PG to produce various traditional products, such as cement retarder, building gypsum and so on, has become mature, and has reached a certain scale of production, the total utilization rate of PG is still very low. The main reason is the large investment and low efficiency. The high value utilization of PG is still being widely studied, with a starting point of the structure of calcium sulfate crystals. Research found that calcium sulfate crystals had three structures: CaSO_4_·2H_2_O, CaSO_4_·0.5H_2_O and CaSO_4_, with the same structural elements: [–Ca–SO_4_–Ca–SO_4_–] alternately forming a chain structure; SO_4_^2−^ was connected to two adjacent Ca^2+^ through O^2−^ [85,86,87,88]. CaSO_4_·2H_2_O, commonly known as dihydrate gypsum, belongs to the monoclinal crystal system. Ca^2+^ binding SO_4_^2-^ forms a double-structured layer. H_2_O molecules are distributed between the double-structured layers, parallel to (010), and the intrinsic structure is flat and lamellar. CaSO_4_·0.5H_2_O, commonly known as hemihydrate gypsum, belongs to the monoclinic crystal system. In the direction of the c axis, Ca^2+^ and SO_4_^2−^ form chains alternately and form a channel with diameter of 4Å. Crystalline water is distributed in the channel, and the structure is resemble short rods. Based on the structure-activity relationship, CaSO_4_·0.5H_2_O is easy to form into whiskers and is used for paper making, plastics, rubber, ceramics, etc. [89,90]. CaSO_4_, commonly known as anhydrous gypsum, has an orthonormal crystal system with alternating chains of Ca^2+^ and SO_4_^2−^ in the direction of the c axis, and its intrinsic structure is granular. CaSO_4_ is stable and insoluble in acids and bases, making it a good filler for composite materials [91].

In recent years, with the development of science and technology, PG has become a research hotspot in the preparation of nanometer calcium sulfate whiskers, nanometer calcium carbonate, sulfur, thiourea and other high-end products. 

**Figure 3:**
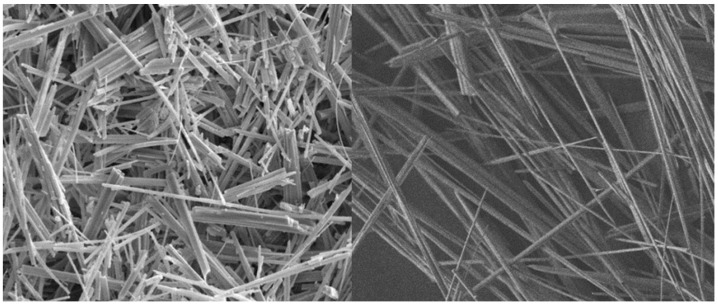
The presentation of calcium sulfate whiskers prepared using PG.

The following is a summary of the studies on the preparation of calcium sulfate whiskers using PG. This is then applied to the effect of composite materials, as shown in Figure 3.

Calcium sulfate whiskers, also known as gypsum whiskers, are loose, needle-like white fibers with an aspect ratio of 30 to 70 and an average length of 30 to 150 microns [92]. There are three types of calcium sulfate whiskers, including no crystal whiskers, semi-crystal whiskers and two-crystal whiskers [93,94]. Calcium sulfate whiskers have the advantages of high strength, chemical corrosion resistance, high temperature resistance, good toughness, strong affinity with rubber and other polymers, and has a stable performance. They can be widely used in rubber, plastic, friction materials, coatings, paint, paper, catalysis, conductive powder and environmental engineering industries and in green environmental protection materials [95,96,97,98]. The main methods of producing calcium sulfate whiskers are by hydrothermal and atmospheric pressure acidification methods [99,100]. The hydrothermal method has been widely used in the controlled preparation of one-dimensional nanomaterials including nanowires, nanorods and nanoribbons using calcium sulfate whiskers. In the preparation of whisker materials by hydrothermal methods, the controlled preparation of crystal morphology can be achieved by controlling the physical and chemical environment.

Research on calcium sulfate whiskers first appeared in the United States and Germany, and calcium sulfate whiskers were first synthesized in 1976. The 1976 U.S. patent US3961105 introduced the method of preparation of calcium sulphate whiskers [101]: mix dihydrate calcium sulphate with water into a paste, solid containing 2–30 g/L, put the suspension in a high pressure reaction kettle, at 105 °C to 150 °C. This results in calcium sulfate hemihydrate whiskers 0.5–5 microns in diameter, with a length to diameter ratio of 6–100. In the same year, German patent DE2613651 also introduced a preparation method of calcium sulfate whiskers under acidic conditions [102]: mix calcium sulfate with water, adjust the pH value to about 3, and react at 120–150 °C hydrothermally for a period of time to obtain calcium sulfate whiskers with a diameter of 1–2 microns and a length of more than 100 microns, and then calcine at 200–800 °C to obtain anhydrous calcium sulfate whiskers.

The research on calcium sulfate whiskers started relatively late in China, starting at the end of the 1980s; however, over the years, research has also come a long way. Liwu et al. [103] took gypsum dihydrate with calcium sulfate content more than 95% as raw material. The ratio of gypsum to water was 1:5–20, the hydrothermal reaction temperature was 70–250 °C, the reaction pressure was 0.26–0.484 MPa, the reaction time was 0.75–10 h, the length of the synthesized calcium dihydrate sulfate whisker was 10–200 μm, and the diameter was 0.1–4 μm. After high temperature roasting, anhydrous calcium sulfate whiskers were obtained, and the morphology of the whiskers was relatively uniform. However, because the product was calcium sulfate dihydrate whisker, the mechanical properties and stability were poor, and the whisker morphology would be destroyed when the temperature exceeds 120 °C. Feng Xiaohua et al. [104] took calcium sulfate dihydrate and calcium sulfate semi-hydrate as raw materials and mixed them with water to form a suspension with a mass fraction of 10%. They put them in a hydrothermal kettle with a stirring speed of 150 r·min^−1^ and reacted at 115 °C and 2 MPa. Finally, calcium sulfate whiskers with a length of 20–50 microns and a diameter of 2–5 microns were prepared. Shi Peiyang et al. [105] prepared calcium sulfate whiskers from PG by the hydrothermal synthesis method and obtained the optimal preparation process through their research: reaction temperature was 140 °C, reaction time was 120 min, solid-liquid ratio was 1:10, initial pH value was 5, raw material particle size was 1.36 microns, and whisker aspect ratio was 82.57. Yuan Zhitao et al. [106] prepared calcium sulfate whiskers with an average diameter of 0.19 microns and a length-diameter ratio of 98 with finely ground gypsum (18.1 microns) as raw material with a solid content of 5% and an initial pH value of 9.8–10.1 at 120 °C under hydrothermal conditions. They believed that the growth process of calcium sulfate whiskers was essentially a process of “solubilization, crystallization and dehydration”. During the growth process, the pH value of the solution, the solid content of the slurry and the particle size of the raw material would all affect the supersaturation in the solution, thus changing the growth rate of the crystal. Cui Yishun [100] prepared calcium sulfate whiskers with an average aspect ratio of 95 using PG and sulfuric acid as raw materials through filtration, heating, dissolution, thermal filtration and other procedures. Optimum process conditions were: reaction temperature 103 °C, reaction time 30 min, stirring speed 280 r·min^−1^, mass ratio of raw material PG, sulfuric acid and water 1:4.6:35. Under these conditions, the whisker yield was 31.6% and the purity was 93.6%. Xu Aiye et al. [107] took PG containing CaCO_3_ impurities as raw materials. They first used H_2_SO_4_ pretreatment of the raw material to convert CaCO_3_ into CaSO_4_, and then prepared calcium sulfate whiskers with a length of about 200~300 μm and a diameter of 1~5 μm under the condition of 3.0~10.0% solids, hydrothermal temperature 110~150 °C and hydrothermal time 1~6 h. Jonestone et al. [108] heated a calcium sulfate slurry in dihydrate with a mass fraction of 20~25% in a continuous stirring reactor at a temperature range of 50~60 °C for a period of time, and obtained flake calcium sulfate crystals in dihydrate with a length of 100~450 μm, a width of 10~45μm and a thickness of 1~3 μm. In addition, Gareth, Kuang, Chen et al. [109,110,111] prepared calcium sulfate nanowires with diameters of 0.01~0.1 μm, lengths of 10~100 μm and length-diameter ratios of 50~1000 by a microemulsion method in the presence of lauryl polyether, polyethylene glycol octyl phenyl ether and polyether alcohol.

In the field of application research on calcium sulfate whiskers, Mitsubishi Pencil company [112] mixed calcium sulfate whisker, red pigment, talcum powder, oxidized paraffin, hydroxymethyl cellulose and water at a ratio of 3:2:1.5:2.5:1:10. After drying, the bending strength of the red Pencil core was up to 50 MPa, an increase of 66.7% compared with that before adding. Ma Pengyang et al. [113] used CaSO_4_ whiskers to strengthen a PVC matrix and prepared light and high strength composite materials. At 80 °C, the CaSO_4_ whiskers were wet modified with Na_2_CO_3_ solution. The surface roughness of CaSO_4_ whiskers was increased from 56.8 nm to 115.6 nm by coating nano calcium carbonate on the surface. Finally, the composite materials were prepared with hierarchical CaSO_4_ whiskers, which increased the flexural strength from 86.3 MPa to 113.2 MPa. Thus, it can be seen that the preparation of nanometer calcium sulfate whiskers from PG is an important way to recycle it.

**Figure 4:**
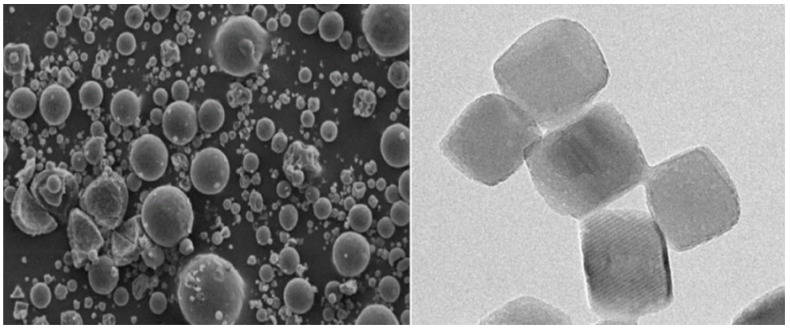
The presentation of nanometer calcium carbonate prepared from PG.

The following is a summary of the studies on the preparation of multiple calcium carbonate products from PG as a Ca resource. As a new kind of functional non-metallic material, nanometer calcium carbonate (Figure 4) has the characteristics of fine particles, large specific surface area and high whiteness.

As a reinforcing filler with good performance and low price, nanometer calcium carbonate is widely used in the industries of paper making, rubber, plastic, ink and medicine. Lu Shangqing et al. [114] used PG as raw material to prepare nano calcium carbonate by reactive crystallization in a gas (CO_2_), liquid (NH_3_·H_2_O) and solid (PG) three-phase system. They found that when the flow rate of carbon dioxide was 251–138 mL/min and the reaction temperature was 30–40 °C, the average particle size of nano calcium carbonate prepared from PG was 86–104 nm; increase of temperature, keeping a relatively low flow rate of carbon dioxide and strictly controlling the reaction time was conducive to the formation of nano CaCO_3_. Baojun Yang et al. [115] used PG as raw material, washed PG and removed impurities by phase transfer, and then prepared ultra-fine light calcium carbonate by a one-step precipitation method. Through research, he determined that the light calcium carbonate prepared under the conditions of precipitation reaction temperature 70 °C, time 75 min, system pH 10 was calcite type calcium carbonate, with an average yield of 95.25%, average purity of 98.17%, and particle size of 20–40 nm.

Other types of calcium carbonate production have used PG as a calcium source, such as calcium carbonate microspheres, highly dispersed calcium carbonate powder, calcite calcium carbonate, etc. In 2019 Baojun Yang et al. [115] created a new route to synthesize calcium carbonate microspheres from PG. Using PG as raw material, sodium gluconate as phase transfer agent and CO_2_ as precipitator, they synthesized monodispersed spherical calcium carbonate by a simple and efficient phase transfer precipitation route at room temperature and atmospheric pressure. They found that the presence of SG inhibited the nucleation and growth of calcite but promoted the formation of vaterite. In 2017, Pengcheng Zhu et al. [116] using PG after removing impurity by desilication, together with ammonium bicarbonate and ammonia as raw materials, researched the effects of reaction temperature, reaction time and stirring speed on the preparation of calcium carbonate. Compared with calcium carbonate produced from PG without desilication, its whiteness and mass fraction were 83% and 97.74%, respectively, increasing by 11% and 13.05%, respectively. In 2016, Zhu Ling et al. [117] took PG as raw material to study the influence of the pH value of the reaction system and other factors on the particle morphology and structure of the calcium carbonate prepared. The results showed that when the pH of reaction solution was ≥11, it was more conducive to the preparation of highly dispersed calcium carbonate powder. In 2014 Liang Yaqin et al. [118] extracted calcium sulfate from PG with ammonium chloride solution, prepared calcite type calcium carbonate in the presence of hydrogen peroxide, and determined the effect of hydrogen peroxide concentration and other conditions on product crystal shape.

It can be seen that the preparation of various types of calcium carbonate using PG as calcium source has realized the efficient utilization of PG.

## 6. Summary and Outlook

As a kind of bulk solid waste material, the impurity removal and high-value utilization of PG is closely related to the sustainable development of resource materials and the protection of the ecological environment. Currently, the comprehensive utilization rate of PG material in China is still far behind that in developed countries, and there is still a lot of work to be done. On the one hand, the existing PG impurity removal methods are mostly for specific impurities. Due to the complex composition of PG with its various impurities, there are some deficiencies and constraints such as incomplete impurity removal, complex experimental processes, high costs, and limited practical application. Therefore, it is necessary to explore novel ways for comprehensive, continuous and efficient removal of PG impurities, laying a foundation for large-scale clean and high value utilization of PG material. On the other hand, for the production of nanometer calcium sulfate whiskers, nanometer calcium carbonate and other high-value products produced from PG, we should focus on thinking about the bottleneck problems existing in the process of large-scale industry applications and exploring solutions to increase the product value in practice and enhance the economic benefits of the comprehensive utilization of the PG material.

## Data Availability

Not applicable.
